# Renal Outcomes in People With HIV-1 and Renal Impairment Treated With Bictegravir/Emtricitabine/Tenofovir Alafenamide: Integrated Analysis From 9 Phase 3/3b Clinical Trials

**DOI:** 10.1093/ofid/ofag265

**Published:** 2026-05-04

**Authors:** Frank A Post, David Wohl, Geoffroy Liegeon, Indira Brar, Debbie Hagins, Yazdan Yazdanpanah, Anchalee Avihingsanon, Hui Liu, Keith Aizen, Jason T Hindman, Samir Gupta

**Affiliations:** Department of Inflammation Biology, School of Immunology & Microbial Sciences, King's College Hospital NHS Foundation Trust, London, UK; Division of Infectious Diseases, Department of Medicine, University of North Carolina, Chapel Hill, North Carolina, USA; Saint-Louis Hospital, AP-HP, Université Paris Cité, Paris, France; Infectious Disease, Henry Ford Health, Detroit, Michigan, USA; Chatham CARE Center, Savannah, Georgia, USA; Bichat–Claude Bernard Hospital, AP-HP, Paris, France; HIV-NAT, Thai Red Cross AIDS Research Centre, Bangkok, Thailand; Clinical Development, Gilead Sciences, Inc., Foster City, California, USA; Clinical Development, Gilead Sciences, Inc., Foster City, California, USA; Clinical Development, Gilead Sciences, Inc., Foster City, California, USA; Division of Infectious Diseases, Department of Medicine, Indiana University School of Medicine, Indianapolis, Indiana, USA

**Keywords:** B/F/TAF, chronic kidney disease, HIV-1, renal impairment, tenofovir alafenamide

## Abstract

Among 1069 people with HIV-1 and mild to moderate renal impairment at baseline treated with bictegravir/emtricitabine/tenofovir alafenamide (B/F/TAF) across 9 clinical trials, renal function remained generally stable over follow-up of up to 240 weeks. Renal adverse events were infrequent, and discontinuation due to renal toxicity was rare.

The global population of people with HIV (PWH) is aging, leading to a rising burden of age-associated comorbidities, including chronic kidney disease (CKD). Chronic kidney disease is particularly common among PWH and is expected to become increasingly prevalent as life expectancy improves [1, 2]. Long-term exposure to antiretroviral therapy (ART), while essential for viral suppression, may contribute to renal impairment, underscoring the importance of evaluating the renal safety profiles of modern ART regimens [2].

Tenofovir alafenamide (TAF)-based regimens have demonstrated improved renal safety compared with tenofovir disoproxil fumarate (TDF)-based therapies [3], although earlier comparisons were frequently conducted using boosted regimens, which may have influenced observed renal and bone outcomes [3, 4]. Bictegravir/emtricitabine/tenofovir alafenamide (B/F/TAF) is a guideline-recommended, single-tablet regimen with proven efficacy and favorable renal and bone safety in the general HIV population [1, 3, 4]. However, long-term renal outcomes in individuals with preexisting mild to moderate renal impairment have not previously been systematically evaluated across extended durations of follow-up.

This integrated analysis pooled data from 9 Phase 3/3b trials to evaluate the long-term renal safety of B/F/TAF in treatment-naive or virologically suppressed adults with a baseline Cockcroft–Gault creatinine clearance (CrCl_CG_) ≥ 30 to <90 mL/min [5–13].

## METHODS

This pooled analysis included data from 9 Phase 3/3b clinical trials of B/F/TAF in adults with HIV-1 and baseline mild to moderate renal impairment (CrCl_CG_ ≥ 30 to <90 mL/min). Participants were treatment naive (GS-US-380-1489, NCT02607930; GS-US-380-1490, NCT02607956; GS-US-380-4458, NCT03547908) [5, 7, 14] or virologically suppressed (GS-US-380-1844, NCT02603120; GS-US-380-1878, NCT02603107; GS-US-380-1961, NCT02652624; GS-US-380-4030, NCT03110380; GS-US-380-4580, NCT03631732; GS-US-380-4449, NCT03405935) [6, 8–10, 12–14] and received B/F/TAF once daily for up to 240 weeks (ie, All B/F/TAF Safety Analysis Set). Participants with baseline CrCl_CG_ ≥ 90 mL/min (no renal impairment) were also included for contextual comparison (Supplementary Tables 1–3) All studies were conducted in accordance with the Declaration of Helsinki and applicable regulatory requirements. Institutional review boards at each site granted ethical approval, and all participants provided written informed consent [5–13].

The primary outcomes were CrCl_CG_ over time and change from baseline. Secondary outcomes included the incidence and type of renal treatment-emergent adverse events (TEAEs) and treatment discontinuation due to renal TEAEs. Adverse events (AEs) were coded using MedDRA version 27.0, with renal AEs defined by the narrow scope of the Standardized MedDRA Query (SMQ) for acute renal failure. Descriptive statistics were used to summarize baseline characteristics, renal function, and TEAEs. Cockcroft–Gault creatinine clearance changes were reported as median and interquartile range (Q1, Q3) at scheduled visits through Week (W) 240.

Participants with renal impairment were stratified by baseline renal function: Stage 2 (CrCl_CG_ ≥ 60 to <90 mL/min), Stage 3a (CrCl_CG_ ≥ 45 to <60 mL/min), and Stage 3b (CrCl_CG_ ≥ 30 to <45 mL/min). Stages 3a and 3b were combined for primary descriptive analyses given the small number of Stage 3b participants (n = 8); Stage 3b results are reported separately in the supplement as exploratory. A comparative analysis of renal outcomes through W144 was performed between B/F/TAF and abacavir/dolutegravir/lamivudine (ABC/DTG/3TC) in studies 1489 and 1844 combined, which had the longest randomized follow-up available for both regimens, and was not intended to support comparative effectiveness conclusions. Exploratory sensitivity analyses of CrCl_CG_ stratified by prior antiretroviral regimen are presented in Supplementary Tables 4 and 5.

## RESULTS

A total of 1069 participants with baseline CrCl_CG_ 30–89 mL/min received B/F/TAF across 9 clinical trials. The median age was 53 years (Q1, Q3: 44, 60), 31% were female, and 51% were White. Eight percent were treatment naive. Most participants (975; 91.2%) had baseline Stage 2 renal function, while 94 (8.8%) had Stage 3a or 3b renal function (CrCl_CG_ ≥ 30 to <60 mL/min; Stage 3a n = 86, Stage 3b n = 8 [exploratory]) (Supplementary Table 1).

**Figure 1.**
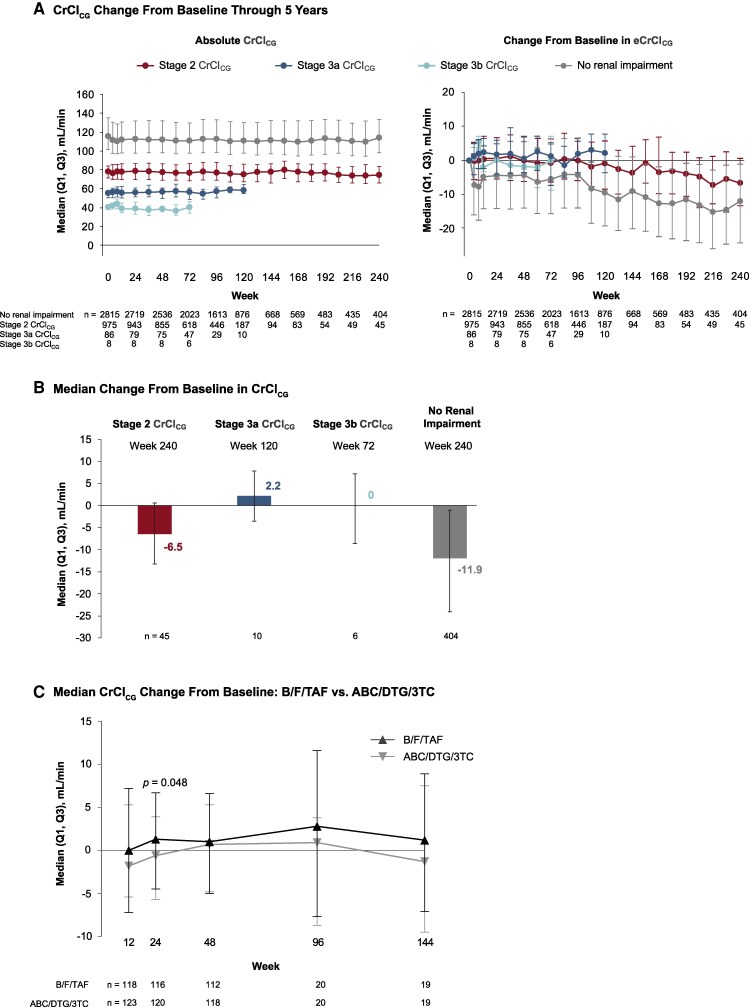
Renal outcomes over time by baseline renal impairment stage in people with HIV-1 treated with B/F/TAF. *A*, Median CrCl_CG_ (left panel) and change from baseline (right panel) through Week 240. *B*, Median change from baseline in CrCl_CG_ at Week 120 by baseline renal function stage. Stage 3b (CrCl_CG_ 30–44 mL/min) is excluded; follow-up data were available through Week 72 only (n = 6; see *A*). *C*, Median CrCl_CG_ change from baseline: B/F/TAF versus ABC/DTG/3TC. ABC/DTG/3TC, abacavir/dolutegravir/lamivudine; B/F/TAF, bictegravir/emtricitabine/tenofovir alafenamide; CrCl_CG_, creatinine clearance by Cockcroft–Gault equation; Q, quartile.

Renal function remained stable over the course of treatment. At W96, the median change in CrCl_CG_ was +0.6 mL/min overall (Q1, Q3: −5.4, 6.6), +0.1 mL/min in Stage 2 participants (Q1, Q3: −5.8, 6.6), and +2.0 mL/min in those with Stage 3a/3b renal function combined (Q1, Q3: −3.6, 5.7). At W120, the median CrCl_CG_ change was −9.4 mL/min (Q1, Q3: −19.2, 1.8) in participants without baseline renal impairment, −0.9 mL/min (Q1, Q3: −8.4, 5.8) in Stage 2, and +2.2 mL/min (Q1, Q3: −3.5, 7.8) in Stage 3a (Figure 1*B*). Longer-term follow-up through W240 showed a modest decline in Stage 2 of −6.5 mL/min (Q1, Q3: −13.2, 0.6), with a similar pattern in those with normal baseline renal function (Figure 1*A*). Stage 3b follow-up was limited to W72 (n = 6); no unexpected patterns were observed at that timepoint, though the small sample size precludes meaningful inference (Figure 1*A*).

In a prespecified comparison of B/F/TAF versus ABC/DTG/3TC (studies 1489 and 1844 combined, CrCl_CG_ 30–89 mL/min), the median change in CrCl_CG_ at W48 was +1.0 mL/min (Q1, Q3: −5.0, 6.6) with B/F/TAF and +0.7 mL/min (−4.8, 5.3) with ABC/DTG/3TC (*P* = .68). A transient difference favoring B/F/TAF was observed at W24 (+1.3 vs −0.6 mL/min; *P* = .048) but was not sustained at W48 or through W96 (Figure 1*C*). Sensitivity analyses stratified by prior regimen revealed no between-group differences in CrCl_CG_ (Supplementary Table 4).

Renal TEAEs were reported in 28 participants (2.6%) with baseline renal impairment. The most common were acute kidney injury (n = 10) and decreased CrCl_CG_ (n = 6) (Supplementary Table 2). One participant (<0.1%) discontinued due to an acute kidney injury assessed as unrelated to B/F/TAF. No cases of proximal renal tubulopathy or Fanconi syndrome were observed. The incidence of renal TEAEs was consistent across studies and within the range reported for comparator integrase strand transfer inhibitor (INSTI)-based regimens [5–13].

## DISCUSSION

This pooled analysis of 9 clinical trials supports a favorable renal safety profile for B/F/TAF in people with HIV-1 and mild to moderate renal impairment. Median changes in CrCl_CG_ were minimal across all renal subgroups [5, 9] and similar to those receiving ABC/DTG/3TC.

The comparison with ABC/DTG/3TC was included to contextualize renal function trajectories rather than to imply comparative effectiveness or contemporary clinical preference. While ABC-containing regimens are now used less frequently in routine practice [15], alternative strategies that avoid both tenofovir prodrugs and abacavir, including dual-therapy regimens, such as dolutegravir/lamivudine, are used in clinical practice for appropriately selected individuals [16].

A modest median decline in CrCl_CG_ was observed through W240 in participants with Stage 2 renal impairment, with a somewhat larger apparent decline in those with normal baseline renal function. The difference likely reflects INSTI-associated inhibition of tubular creatinine secretion (more pronounced at higher baseline CrCl_CG_) and potential attenuation of CrCl_CG_ decline in the renal impairment group through switching from TDF- to TAF-containing regimens [17]; neither represents a true reduction in glomerular filtration rate. Exploratory subgroup analyses showed generally similar CrCl_CG_ trajectories by baseline antiretroviral regimen (Supplementary Table 4). While longer-term data were limited in those with Stage 3a and 3b renal impairment, no unexpected patterns were observed, acknowledging the limited sample size [8].

The incidence of renal TEAEs was low, affecting 2.6% of participants with renal impairment. No instances of proximal renal tubulopathy or Fanconi syndrome were observed, toxicities historically associated with TDF and not observed with TAF [4]. Discontinuation due to renal TEAEs was rare, with only 1 participant (<0.1%) withdrawing due to an acute kidney injury assessed as unrelated to B/F/TAF.

Strengths of this analysis include its long follow-up duration (up to 5 years), large pooled study population, and inclusion of a comparator non–TAF treatment group (ABC/DTG/3TC) [7, 9]. Although sex and race distributions were reasonably representative, the pooled trials included limited representation of people with HIV from low- and middle-income countries, which may limit generalizability to global populations. This pooled analysis included participants from multiple clinical trials with heterogeneous study populations, including both ART-naive individuals and those who were virologically suppressed on prior regimens, which may influence renal outcome trajectories. The number of participants with Stage 3b renal impairment was small, limiting generalizability in that subgroup. Renal function was assessed using only the Cockcroft–Gault equation, without confirmation from cystatin C–based measurements or other alternative estimated glomerular filtration rate (eGFR) estimations. Furthermore, creatinine-based CrCl estimates may be modestly influenced by integrase inhibitor–associated inhibition of tubular creatinine secretion, which can result in small increases in serum creatinine without reflecting true changes in glomerular filtration. Any resultant misclassification of CKD stage—for example, apparent Stage 2 participants whose true GFR would place them in Stage 1—would be expected to be small in magnitude and would not materially alter the overall conclusions regarding the renal safety of B/F/TAF in this population.

In conclusion, this pooled analysis of 9 Phase 3/3b clinical trials provides long-term descriptive data on renal function among people with HIV-1 and mild to moderate renal impairment treated with B/F/TAF. Renal function was generally stable across all renal impairment strata over follow-up periods up to 240 weeks. The low incidence of renal-related adverse events and absence of proximal tubulopathy or Fanconi syndrome are consistent with the established renal safety profile of TAF.

## Supplementary Material

ofag265_Supplementary_Data

## Supplementary Data

Supplementary materials are available at *Open Forum Infectious Diseases* online. Consisting of data provided by the authors to benefit the reader, the posted materials are not copyedited and are the sole responsibility of the authors, so questions or comments should be addressed to the corresponding author.
