# Supplementary material for: Renal Outcomes in People With HIV-1 and Renal Impairment Treated With Bictegravir/Emtricitabine/Tenofovir Alafenamide: Integrated Analysis From 9 Phase 3/3b Clinical Trials
Source: Open Forum Infect Dis. 2026 May 4;13(5):ofag265. doi: 10.1093/ofid/ofag265 (PMC13192347; doi:10.1093/ofid/ofag265)
Supplement: ofag265_Supplementary_Data [file ofag265_supplementary_data.pdf]

Supplementary Table S1. Baseline Demographics and Disease Characteristics by Baseline $CrCl_{CG}$

Characteristic	≥30 to <90 mL/min (RI)				≥90 mL/min (No RI, n=2815)
	≥30 to <45 mL/min (Stage 3b, n=8)	≥45 to <60 mL/min (Stage 3a, n=86)	≥60 to <90 mL/min (Stage 2, n=975)	Overall (n=1069)	
**Age, years, median (Q1, Q3)**	70 (61, 75)	62 (54, 67)	52 (43, 59)	53 (44, 60)	39 (31, 49)
**Female sex at birth, n (%)**	2 (25)	34 (40)	296 (30)	332 (31)	642 (23)
**Race, n (%)[a]**					
White	5 (63)	45 (52)	492 (51)	542 (51)	1374 (49)
Black	2 (25)	33 (38)	321 (33)	356 (33)	998 (35)
Asian	0	5 (6)	110 (11)	115 (11)	240 (9)
Other[b]	1 (13)	3 (3)	52 (5)	56 (5)	203 (7)
**Hispanic or Latine ethnicity, n (%)**	1 (13)	12 (14)	148 (15)	161 (15)	529 (19)
**$CrCl_{CG}$, mL/min, median (Q1, Q3)**	40.8 (38.6, 42.5)	55.8 (51.0, 58.2)	78.6 (72.0, 84.8)	77.7 (69.6, 84.6)	116.8 (102.7, 136.8)
**Treatment history, n (%)**					
Treatment-naïve	0	5 (6)	79 (8)	84 (8)	670 (24)
Virologically suppressed	8 (100)	81 (94)	896 (92)	985 (92)	2145 (76)

[a]Percentages may not add to 100 due to rounding; [b]Includes American Indian or Alaska Native; Native Hawaiian or Other Pacific Islander; other race; and participants with Not Permitted designation (when local regulators did not allow collection of race/ethnicity information). $CrCl_{CG}$, creatinine clearance by Cockcroft-Gault equation; Q, quartile; RI, renal impairment.

Supplementary Table S2. Renal TEAEs and Discontinuation of B/F/TAF Due to Renal TEAEs

	$CrCl_{CG}$ ≥30 to <90 mL/min (RI), n=1069	$CrCl_{CG}$ ≥90 mL/min (No RI), n=2815
**Any renal TEAE, n (%)**	28 (2.6)	71 (2.5)
Acute kidney injury	10 (0.9)	14 (0.5)
Creatinine renal clearance decreased	6 (0.6)	5 (0.2)
Proteinuria	5 (0.5)	39 (1.4)
Blood creatinine increased	4 (0.4)	8 (0.3)
Renal impairment	4 (0.4)	0
Renal failure	2 (0.2)	4 (0.1)
Glomerular filtration rate decreased	1 (<0.1)	0
Protein urine present	0	4 (0.1)
Albuminuria	0	2 (<0.1)
Blood creatinine abnormal	0	1 (<0.1)
Blood urea increased	0	1 (<0.1)
Urine output decreased	0	1 (<0.1)
**Renal TEAE leading to B/F/TAF discontinuation, n (%)**	1 (<0.1)	0
Acute kidney injury (leading to discontinuation)	1 (<0.1)	0

B/F/TAF, bictegravir/emtricitabine/tenofovir alafenamide; $CrCl_{CG}$, creatinine clearance by Cockcroft-Gault equation; RI, renal impairment; TEAE, treatment-emergent adverse event.

Supplementary Table S3. Summary of Included Trials for Baseline Regimen Subgroup Analysis

Study	N	Median Age (y)	Female (%)	White (%)	Black (%)	Asian (%)	Treatment History
**380-1489**	568	34	10.2	57.2	36.7	2.5	TN (n=314)<br>ABC/DTG/3TC (n=254)
**380-1490**	320	33	12.5	57.2	30.3	2.2	TN (n=320)
**380-4458**	210	33	5.2	4.8	2.9	91.4	TN (n=121)<br>DTG+F/TDF (n=89)
**380-1844**	547	47	10.8	73.0	21.3	3.3	VS<br>ABC/3TC-containing (n=547)

**380-1878**	534	48	17.6	65.4	26.4	2.4	VS<br>TDF-containing (n=454)<br>ABC/3TC-containing (n=80)
**380-1961**	219	38	100.0	33.3	42.9	15.1	VS<br>TDF-containing (n=219)
**380-4030**	90	51	10.0	73.9	25.0	1.1	VS<br>TDF-containing<br>(n=90)
**380-4449**	7	67	14.3	100.0	0	0	VS<br>TDF-containing<br>(n=7)
**380-4580**	158	50	31.6	0	92.4	0	VS<br>ABC/3TC-containing (n=68)<br>TDF-containing (n=90)

Participants in subgroup of ABC/3TC-Containing Regimen or Treatment Naive to B/F/TAF (N=1704) or subgroup of TDF Containing Regimen to B/F/TAF (N= 949) are included.

N with renal impairment (baseline $CrCl_{CG}$ 30–89 mL/min) from studies contributing to sensitivity analysis; studies without annotated RI N contributed participants primarily in CKD Stage 2 stratum within the combined analyses.

CKD Stage 2 (baseline $CrCl_{CG}$ 60–89): N=294; CKD Stage 3a (baseline $CrCl_{CG}$ 45–59): N=28; CKD Stage 3b: none from this study.

CKD Stage 2 (baseline $CrCl_{CG}$ 60–89): N=238; CKD Stage 3a (baseline $CrCl_{CG}$ 45–59): N=23; CKD Stage 3b: n=1.

ABC, abacavir; ART, antiretroviral therapy; B/F/TAF, bictegravir/emtricitabine/tenofovir alafenamide; $CrCl_{CG}$, creatinine clearance by Cockcroft-Gault equation; DTG, dolutegravir; F/TDF, emtricitabine/tenofovir disoproxil fumarate; 3TC, lamivudine; TDF, tenofovir disoproxil fumarate; TN, Treatment-naïve; virologically suppressed, VS.

Supplementary Table S4. $CrCl_{CG}$ Trajectories by Baseline Regimen Subgroup Sensitivity Analysis

Subgroup	ABC/3TC or Tx-naïve → B/F/TAF<br>Median (Q1, Q3)	TDF → B/F/TAF<br>Median (Q1, Q3)	Between-Group p-value (Wilcoxon)
**Baseline $CrCl_{CG}$ 30–89 mL/min (all renal impairment[1])** (ABC/3TC or Tx-naïve: n=372; TDF: n=298)			
$\Delta CrCl_{CG}$ at Week 48, median (Q1, Q3), mL/min	+1.1 (-4.9, 7.9)	+1.8 (-4.6, 10.2)	p=0.23
$\Delta CrCl_{CG}$ at Week 96, median (Q1, Q3), mL/min	+2.2 (-5.9, 7.4)	+1.4 (-4.2, 8.4)	p=0.74
**Baseline $CrCl_{CG}$ 60–89 mL/min (CKD Stage 2)** (ABC/3TC or Tx-naïve: n=344; TDF: n=274)			
$\Delta CrCl_{CG}$ at Week 48, median (Q1, Q3), mL/min	+1.1 (-5.4, 8.1)	+1.5 (-5.4, 10.2)	p=0.39
$\Delta CrCl_{CG}$ at Week 96, median (Q1, Q3), mL/min	+1.8 (-6.0, 7.7)	+0.9 (-4.3, 7.2)	p=0.85
**Baseline $CrCl_{CG}$ 45–59 mL/min (CKD Stage 3a)** (ABC/3TC or Tx-naïve: n=28; TDF: n=23)			
$\Delta CrCl_{CG}$ at Week 48, median (Q1, Q3), mL/min	+1.0 (-1.8, 5.5)	+5.4 (-1.1, 10.8)	p=0.18
$\Delta CrCl_{CG}$ at Week 96, median (Q1, Q3), mL/min	+4.7 (2.4, 7.1)	+9.0 (2.3, 19.8)	p=0.58

[1]Participants with Baseline $CrCl_{CG}$ 30–89 mL/min in subgroup of ABC/3TC-Containing Regimen or Treatment Naive to B/F/TAF or subgroup of TDF Containing Regimen to B/F/TAF are included.

CKD Stage 3b (CrClCG 30–44 mL/min): Only 1 participant in the TDF subgroup had Stage 3b renal function at baseline; formal comparison was not feasible. The $CrCl_{CG}$ in this single participant increased from 42.4 mL/min at baseline to 75.8 mL/min at Week 96, consistent with the overall pattern of renal function improvement observed in the TDF-to-TAF switch population. Median change from baseline in CrClCG (mL/min) at Weeks 48 and 96 is shown for participants stratified by baseline antiretroviral regimen (ABC/3TC-containing or treatment-naïve vs TDF-containing), across all renal impairment strata. Between-group p-values are from the two-sided Wilcoxon rank-sum test.

Supplementary Table S5. Treatment-Emergent Renal Adverse Events by Baseline Regimen Subgroup

Renal TEAE	ABC/3TC or Treatment-naïve → B/F/TAF (n=372), n (%)	TDF → B/F/TAF (n=298), n (%)	Total (n=670), n (%)
**Any renal TEAE**	14 (3.8%)	7 (2.3%)	21 (3.1%)
**Acute kidney injury**	5 (1.3%)	1 (0.3%)	6 (0.9%)
**CrCl decreased**	4 (1.1%)	2 (0.7%)	6 (0.9%)
**Blood creatinine increased**	3 (0.8%)	1 (0.3%)	4 (0.6%)
**Proteinuria**	4 (1.1%)	0	4 (0.6%)
**Renal impairment**	1 (0.3%)	2 (0.7%)	3 (0.4%)
**GFR decreased**	0	1 (0.3%)	1 (0.1%)
**Renal failure**	0	1 (0.3%)	1 (0.1%)

Participants with Baseline $CrCl_{CG}$ 30–89 mL/min in ABC/3TC-Containing Regimen or Treatment Naive to B/F/TAF subgroup or TDF Containing Regimen to B/F/TAF subgroup are included.

Renal TEAEs (by MedDRA preferred term, Acute Renal Failure SMQ narrow scope) in participants with $CrCl_{CG}$ 30–89 mL/min at baseline, stratified by baseline antiretroviral regimen. TEAEs defined as AEs with onset on or after B/F/TAF start date and no later than 30 days after permanent discontinuation, or any AE leading to premature discontinuation. AEs coded using MedDRA 27.0. ABC, abacavir; B/F/TAF, bictegravir/emtricitabine/tenofovir alafenamide; $CrCl_{CG}$, Cockcroft-Gault creatinine clearance; GFR, glomerular

filtration rate; TEAE, treatment-emergent adverse event; TDF, tenofovir disoproxil fumarate. No participant discontinued B/F/TAF due to a renal TEAE in either subgroup
